# Cutaneous Adverse Effects From Diabetes Devices in Pediatric Patients With Type 1 Diabetes Mellitus: Systematic Review

**DOI:** 10.2196/59824

**Published:** 2024-11-07

**Authors:** Alicia Podwojniak, Joseph Flemming, Isabella J Tan, Hira Ghani, Zachary Neubauer, Anne Jones

**Affiliations:** 1 Rowan-Virtua School of Osteopathic Medicine, 113 Laurel Rd, Stratford, NJ, 08084, United States, 1 (856) 566-6789; 2 Rutgers Robert Wood Johnson Medical School, New Brunswick, NJ, United States; 3 Department of Dermatology, Northwestern University Feinberg School of Medicine, Chicago, IL, United States; 4 Sidney Kimmel Medical College of Jefferson University, Philadelphia, PA, United States

**Keywords:** insulin pumps, continuous glucose monitoring, type 1 diabetes, lipohypertrophy, contact dermatitis, lipodystrophy

## Abstract

**Background:**

Continuous glucose monitoring (CGM) and continuous subcutaneous insulin infusions (CSIIs) are the current standard treatment devices for type 1 diabetes (T1D) management. With a high prevalence of T1D beginning in pediatrics and carrying into adulthood, insufficient glycemic control leads to poor patient outcomes. Dermatologic complications such as contact dermatitis, lipodystrophies, and inflammatory lesions are among those associated with CGM and CSII, which reduce glycemic control and patient compliance.

**Objective:**

This systematic review aims to explore the current literature surrounding dermatologic complications of CGM and CSII as well as the impact on patient outcomes.

**Methods:**

A systematic review of the literature was carried out using PRISMA (Preferred Reporting Items for Systematic Reviews and Meta-Analyses) 2020 guidelines using 5 online databases. Included articles were those containing primary data relevant to human participants and adverse reactions to CGM and CSII devices in pediatric populations, of which greater than 50% of the sample size were aged 0‐21 years. Qualitative analysis was chosen due to the heterogeneity of outcomes.

**Results:**

Following the application of exclusion criteria, 25 studies were analyzed and discussed. An additional 5 studies were identified after the initial search and inclusion. The most common complication covered is contact dermatitis, with 13 identified studies. Further, 7 studies concerned lipodystrophies, 5 covered nonspecific cutaneous changes, 3 covered unique cutaneous findings such as granulomatous reactions and panniculitis, and 2 discussed user acceptability.

**Conclusions:**

The dermatologic complications of CGM and CSII pose a potential risk to long-term glycemic control in T1D, especially in young patients where skin lesions can lead to discontinuation. Increased manufacturer transparency is critical and further studies are needed to expand upon the current preventative measures such as device site rotation and steroid creams, which lack consistent effectiveness.

## Introduction

Type 1 diabetes (T1D) is a chronic metabolic disease that results from the autoimmune destruction of pancreatic beta islet cells with subsequent loss of endogenous insulin production. With a growing global incidence, inadequate surveillance of glucose monitoring, dietary management, and insulin injections pose a lifelong threat and burden to patients [1]. Although T1D treatment has improved significantly since the development of exogenous insulin in 1921, the acute risks of hypoglycemia and associated long-term morbidity from poor glycemic control necessitate an imminent need for more sustainable treatment [1]. T1D carries high morbidity, mortality, and poor quality of life [2]. There may be associated profound psychological distress and subsequent poor adherence to treatment [3].

Continuous glucose monitoring (CGM) and continuous subcutaneous insulin infusion (CSII) are currently the standards of care for managing T1D. CGMs are devices that monitor glucose levels within the interstitial fluid of subcutaneous adipose tissue every few minutes, replacing the need for manual finger sticks but requiring device replacement every 1‐2 weeks [4]. CGMs can be used concomitantly with manual exogenous insulin or with automated insulin pumps, which are programmed to dose and release insulin. Closed loop systems allow the CGM and insulin pump to communicate and automatically dose depending on measured glucose levels. Flash glucose monitoring (FGM) require patients to scan their cellular device over the CGM to obtain the data [4]. For CSII devices, infusion set cannulas are inserted subcutaneously, set onto the skin with adhesives, and connected via plastic tubing to the electronic device [2].

Contact dermatitis, local erythematous reactions, infection, and lipodystrophies are among the most commonly reported potential cutaneous side effects from using these devices [5]. Such reactions can lead to discontinued use and reliance on manual insulin administration, which has been shown to be less effective at optimizing glycemic control [4]. Primarily in pediatric patients, in whom tolerance for adverse skin reactions may be reduced, we suspect that identification and subsequent resolution of cutaneous adverse effects will promote increased adherence and optimized glycemic control. This systematic review aims to identify the existing cutaneous adverse reactions related to subcutaneous insulin infusion systems and CGM devices in pediatric patients.

## Methods

This systematic review was conducted using the PRISMA (Preferred Reporting Items for Systematic Reviews and Meta-Analyses) 2020 guidelines using PubMed, Scopus, Embase, Cochrane, and Web of Science databases [6]. This paper is registered on Prospero CRD42023489106. Using the National Library of Medicine Medical Subject Heading to determine the best selection of potential search terms, the following were derived and used: (“insulin infusion System” OR “insulin infusion systems” OR “insulin pump” OR “implantable programmable insulin pump” OR “CGM” OR “continuous glucose monitor”) AND (“skin manifestation” OR “skin” OR “skin reaction” OR “cutaneous manifestation” OR “cutaneous reaction” OR “cutaneous” OR “dermatologic manifestation” OR “dermatologic reaction” OR “dermatologic”) AND (“pediatric” OR “child”). The following inclusion criteria were applied: original articles that involved primary data, that is, randomized controlled trials, retrospective studies, case studies, case series, human-only studies, literature published within the last 5 years (2018‐2023), international studies, and studies about adverse cutaneous reactions to insulin infusion systems in pediatric patients. Exclusion criteria included abstracts, articles lacking full text, studies still in progress, articles that did not include mention of adverse cutaneous reactions to insulin infusion systems in pediatric patients, studies that had less than 50% pediatric patients or a mean age range outside of 0‐21 years.

Duplicate studies following initial retrieval were identified and sorted through by 2 reviewers (AP and JF) to ensure there were no further duplicates. After removing duplicates, the abstracts and titles were screened for the inclusion criteria (AP). After the title and abstract appraisal, 2 reviewers independently conducted a full-text review (AP and JF). The remaining studies then continued to the data extraction phase. The risk of bias was assessed by AP and JF using the Joanna Briggs Institute (JBI) Critical Appraisal Checklist, which allows assessment of risk grading and scoring at low, moderate, or high [7]. Following these steps, data were extracted from the shortlisted articles, focusing on dermatologic reactions as the primary outcome. Secondary outcomes were device adherence and the efficacy of insulin infusion as measured by HbA_1c_. Given the heterogeneity of studies included in the review, a qualitative analytic approach was chosen.

## Results

### Overview

**Figure 1.**
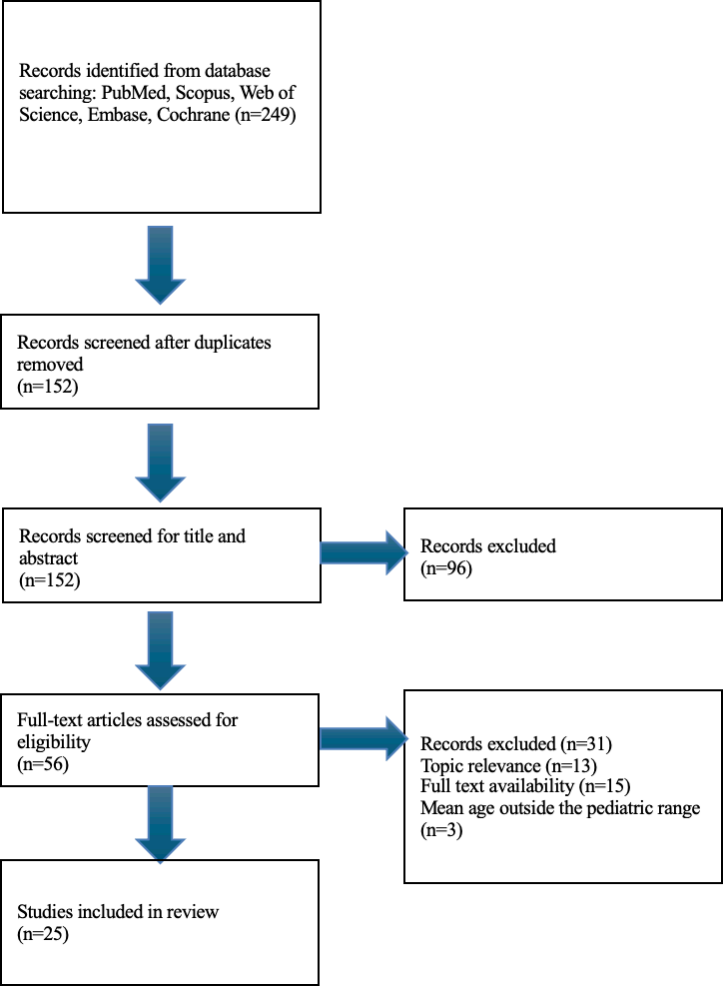
Flow diagram of the systematic study selection process.

The initial search retrieved 249 studies, of which 157 were duplicates (Figure 1). Of the remaining 152 articles, 56 were included in the abstract appraisal, and 96 were excluded due to the article type, wrong patient population, or not being relevant to the topic. Quality full-text appraisal included 25 studies. Of these, the initial search yielded 12 papers discussing contact dermatitis, 6 discussing lipodystrophy, 4 discussing nonspecific cutaneous changes and burden, and 3 describing other unique cutaneous reactions. An additional 5 papers were added to supplement the identified articles, although they were not identified via the initial search terms. A table of findings is summarized in (Table 1) and the basis of levels of study type is outlined according to The Centre for Evidence-Based Medicine Levels of Therapeutic Studies [8].

**Table 1.** Summary of identified studies.

Authors	Cutaneous manifestation	Affected, n (%)	Age (years) unless otherwise stated, mean (SD)	Discontinued use of insulin devices (%)	Glycemic control outcomes	Quality of study^a^
Rigo et al [9]	Nonspecific cutaneous reactions	121 (60)	13.9 (4.8)	22	Not included	2b
Hilliard et al [10]	Nonspecific cutaneous reactions	55 (nonspecific)	5 (1.5)	Not included as a measure specific to cutaneous reaction	Not included	2b
Genève et al [11]	Nonspecific cutaneous reactions	198 (33.8)	11.75 (3.84)	4.3	Not included	2b
Messaaoui et al [12]	Nonspecific cutaneous reactions	334 (not available)	13.6 (not available)	Not included as a measure specific to cutaneous reaction	Not included	2b
Sørensen et al [13]	Ultrasound determined subcutaneous changes	161 (not applicable)	11 (not available)	Not included	No effect of hyperechogenicity (an indicator of lipohypertrophy) on HbA_1c_	2b
Ahrensbøll-Friis et al [14]	Contact dermatitis	30 (100)	13.8 (12.7)	Not included	Not included	2b
Alves da Silva et al [15]	Contact dermatitis	15 (100)	9.3 (3.5)	26% discontinued device and switched to another, 0% totally discontinued use of any device	Not included	2b
Lombardo et al [16]	Contact dermatitis	139 (56)	11.1 (3.3)	0.01	Not included	2b
Herman et al [17]	Contact dermatitis	12 (100)	11.5 (4)	16	Not included	2b
Huang and DeKoven [18]	Contact dermatitis	1 (100)	11 (not applicable)	Not included	Not included	4
Enberg et al [19]	Contact dermatitis	1 (100)	6 (not applicable)	Discontinued use and changed brands	Not included	4
Lyngstadaas et al [20]	Contact dermatitis, systemic dermatitis, and infection	1 (100)	8 (not applicable) months	Discontinued use and changed brands	Not included	4
Cichoń et al [21]	Contact dermatitis	1 (100)	15 (not applicable)	Not included	Not included	4
Ulriksdotter et al [22]	Contact dermatitis	2 (100)	8 (not applicable), 10 (not applicable)	Discontinued use and changed brands	Not included	4
Svedman et al [23]	Contact dermatitis	8 (100)	8 (not applicable)	Discontinued use and changed brands prior to study	Not included	2b
Passanisi et al [24]	Contact dermatitis	21 (100)	12.1 (3.7)	38.1% discontinued use	No significant change in glycemic control as measured by HbA_1C_	2b
Mowitz et al [25]	Contact dermatitis	4 (100)	9.75 (not available)	75% discontinued use or switched brands	Not included	4
Demir et al [26]	Lipohypertrophy	254 (17.1)	14.9 (4.7)	Not included	Nonsignificant changes increased HbA_1C_ associated with lipohypertrophyIncreased number of hypoglycemic episodes for those with lipohypertrophy (*P*=.007)	2b
Lombardo et al [27]	Lipohypertrophyand lipoatrophy	151(lipohypertrophy 44.3 andlipoatrophy 0.9)	11.9 (4.7)	Not included	Difference in correlation variation (*P*<.05) and blood glucose SD score (*P*=.02) among patients with lipodystrophies	2b
Vitebskaya et al [28]	Contact dermatitisand lipohypertrophy	50(contact dermatitis 45 andlipohypertrophy 63)	12 (not available)	Not included	Not included	2b
Burgmann et al [29]	General dermatologic complication and lipohypertrophy	369 (general dermatologic complication 91.8) and 369 (lipohypertrophy 46.8)	12.3 (4.4)	0% discontinued use	Increased HbA_1c_ in those with lipohypertrophy (*P*=.02)	2b
Deeb et al [30]	Lipohypertrophy	104 (39)	12.11 (4.1)	Not included	Increased HbA_1c_ in those with lipohypertrophy (*P*<.001)	2b
Xatzipsalti et al [31]	Lipoatrophy	2 (100)	6 (not applicable), 9 (not applicable)	Insulin-induced, changed insulin types without improvement	Not included	4
Kordonouri et al [32]	Lipoatrophy	14 (100)	14.7 (not available)	Not included	Nonsignificant changes in HbA_1c_	1b
Perez et al [33]	Granulomatous reaction	1 (100)	6 (not applicable)	Switch from CSII^b^ to multiple daily injection improved lesions	Not included	4
Smith et al [34]	Panniculitis reaction	1 (100)	13 (not applicable)	Not included	HbA_1C_ rise from 7.2% to 12.5% following development of nodules	4
Edwards et al [35]	Panniculitis	1 (100)	17 (not applicable)	Multiple changes trialed and failed	Not included	4
Engler et al [36]	User acceptability	114 (not applicable)	10.7 (3.8)	Not included	Not included	2b
Al Hayek et al [37]	User acceptability	67 (not available)	13 (not available) to 19 (not available)	Not included	Not included	2b

a From the Centre for Evidence-Based Medicine [8].

b CSII: continuous subcutaneous insulin infusion.

### Nonspecific Cutaneous Outcomes

In total, 2 qualitative surveys report generalized skin complaints as barriers to using CGMs and CSII devices [9,10]. Increased complications were seen in those who used both devices rather than just 1 (69% vs 39%). Erythema, pruritus, pain, rash, skin change, infection, and existing skin condition exacerbation were the most commonly self-reported complications in descending order [9]. Further, 22% (16/72) of respondents reported discontinuing the use of the devices as a result of these complications, and only 7% (5/72) reported visiting a dermatologist to manage these complications. Genève et al [11] reported 33.8% (67/198) reported skin reactions, with reactions in 30.4% (45/198) of those who used CSII and 23.5% (46/198) of those using CGM devices. Erythema (89.6%) (60/67), itching (82.1%) (55/67), presence of vesicles (35.8%) (24/67), and squamous lesions (26.9%) (18/67) were most commonly reported [11]. Detrimental consequences of these lesions included irregular usage (21.9%), device discontinuation (4.3%), device model change (13.1%), school absences (10.9%), sleep disturbance (35.5%), and discontinuing hobbies (13.2%) [11].

Sørenson et al [13] investigated the subcutaneous changes, including echogenicity, vascularity, and device distance via ultrasound, resulting from 1 year of device usage. Subcutaneous hyperechogenicity frequency, a measure of lipohypertrophy, and vascularization increased significantly over time for CSII devices (*P*<.001 and *P*=.009) but not for CGM. Subcutaneous hyperechogenicity did not predict poor glycemic control by HbA_1c_ in this study (*P*=.11) [13].

It was also noted that among patients using FGM, adverse events were more frequently reported compared to those using self-monitoring of blood glucose. These included premature sensor losses (31.8% vs 12.4%; *P*=.001), skin reactions (18.2% vs 2.6%; *P*<.001), and local pain (6.8% vs 0%; *P*<.001) [12].

### Allergic Contact Dermatitis

In total, 7 studies and 6 case reports describe allergic contact dermatitis (ACD) with various identified culprit allergens. Most cases were due to tapes and adhesives, and many others were attributable to allergens within the housing of the pump or sensor [14,15]. Isobornyl acrylate (IBOA) was identified as the primary culprit allergen, with positive patch testing results in 4 studies [14,15,17,23]. Abitol, colophonium [14,16] benzoyl peroxide [14,15], N,N-dimethylacrylamide, colophonium, sesquiterpene lactone, and various acrylates [17,23], were also identified as contributors in a variety of device types and brands. A wide variety of commonly used devices were used. There was some overlap regarding brand and product type (adhesive, plastic, plaster, and CGM or CSII). Many patients had often used and failed at least 1 or 2 other devices with various compositions, suggesting cross-reactivity among products and brands [23]. Additional reactions include pruritus, fluid leakage, hyperpigmentation, bleeding, infection, and scarring, which were treated with topical corticosteroids and moisturizers [15]. Hypoallergenic bandage barrier use was the most reported solution to minimize the reaction, with a 43.7% improvement in 1 study [16]. Additional prevention measures were hydrocolloid and silicone-based plaster barriers, topical steroids, topical antibiotics, emollient creams, and topical antihistamines [24].

Further, 5 studies reported the need for complete discontinuation or switching to a different device [15,17,23,30]. This metric was not included in 2 articles [14,16]. Effects on glycemic control were generally not included, except in 2 articles that did not identify a significant difference in HbA_1c_ among patients with or without ACD without commenting on the discontinuation or continuation of devices [16,24].

In total, 5 (n=6) case reports were identified in this review that describe pediatric patients presenting with contact dermatitis from their diabetes devices. Further, 2 (n=3) of these cases describe patients without a history of atopic dermatitis (AD) who developed contact dermatitis reactions from multiple infusion sets and CGMs, with alternating brand use and site placement [21,22,24]. IBOA and other acrylates were identified [21] along with dipropylene glycol diacrylate [22] as culprit allergens. In 2 of these patients, successful switching of devices resolved the lesions [22]. Further, 2 (n=2) cases report the presentation of patients with a history of AD who developed contact dermatitis, in which the first began as an exacerbation of AD [19] and the second progressed to severe, systemic contact dermatitis reaction with subsequent infections requiring hospitalization [20]. IBOA was a contributing allergen in both cases, while dicyclohexylmethane-4,4_0_-diisocyanate [19] and 4-tert-butylcatechol [19,20] were also identified. Discontinuation and switching of devices yielded a positive outcome in 1 case [20] and was not reported in another [19]. The last case describes the development of contact dermatitis from CSII, CGM, and an adhesive barrier wipe used between sensor changes that contained isopropyl alcohol and colophony. Before wipe use, the patient did not react to the devices on their own. The authors suggest a sensitization that occurred due to wiping and progressed with subsequent exposure to the devices, as their patch testing results were positive for IBOA, sesquiterpene lactone, and colophony [18]. It is not reported whether the patient discontinued use because of their reaction.

In 1 case series investigating allergic reactions to the FreeStyle Libre glucose sensor, 7 patients underwent patch testing with IBOA and N,N-dimethylacrylamide [25]. The results revealed sensitization to both IBOA and N,N-dimethylacrylamide in 6 patients, with 1 patient showing a reaction solely to N,N-dimethylacrylamide [25]. Gas chromatography–mass spectrometry analysis confirmed the presence of IBOA in adhesive patches and both IBOA and N,N-dimethylacrylamide in sensor extracts, suggesting that both compounds, commonly found in adhesives of medical devices such as glucose sensors, should be considered during patch testing for suspected allergic reactions [25].

### Lipodystrophies

Several studies examined the incidence of lipodystrophies, including lipohypertrophy and lipoatrophy, from the use of CGMs or CSII devices. Bleeding, bruising, and pain at the injection site were commonly reported regardless of injection type [26]. Rates of lipohypertrophy were significantly higher in the multiple daily injections (MDI) group compared to the CSII group (*P*=.001) [26]. A similar, nonsignificant finding was seen in Vibetskaya et al [28]. In contrast, Burgmann et al [29] found a higher incidence of lipohypertrophy associated with CSII compared to MDI (n=125, 46.8% vs n=44, 42.2) as opposed to the aforementioned studies [26,28]. For those with lipohypertrophy, higher average insulin doses were required to maintain metabolic control (0.97 U/kg/day vs 0.78 U/kg/day), and HbA_1C_ was increased [26] Significantly elevated HbA_1c_ levels were noted in 2 studies (*P*=.02 and *P*<.001) indicating a therapeutic detriment related to the incidence of lipohypertrophy [29,30]. Increased daily insulin usage was not significantly associated with lipohypertrophy [28,30]. The incidence of hypoglycemic episodes was significantly greater in those with lipohypertrophy (*P*=.007) [18]. Incidence of lipohypertrophy was significantly decreased in relation to adequate site rotation (*P*=.02 and *P*=.02) [26,30]. Overall, quality of life impairment was reported as low or absent in 95% of patients regardless of insulin therapy modality [29], and 0 participants discontinued the use of these devices. In a sample of 151 participants, Lombardo identified a prevalence of lipohypertrophy at 44.3% (94/212), and of lipoatrophy 0.9% (2/212). Lipodystrophies were associated with negative consequences in glycemic control [27].

Xatzipsalti et al [31] described 2 cases in which children with lipoatrophy were resistant to standard treatment modalities and experienced regression of lipoatrophy following laser treatment. First, a child aged 6 years was found to have sites of lipoatrophy on the right upper thigh and bilateral buttocks. Lipoatrophy did not improve after switching to insulin glulisine or with the administration of 4% sodium chromoglycate [31]. Due to the failure of conservative treatments, a CO2 laser, which generates a D-pulse that targets deep subcutaneous tissue, was directed at sites of lipoatrophy on the bilateral buttocks [31]. Further, 9 months following treatment, a dramatic reversal of lipoatrophy sites on the buttocks was observed, whereas the lipoatrophy site of the right upper thigh showed little to no improvement where sodium chromoglycate treatment was continued [31]. The same authors further discussed an identical treatment course in a patient aged 9 years [31].

Kordonouri et al [32] conducted a randomized controlled trial to determine the effectiveness of zinc-free insulin formulations in reducing lipoatrophy. All participants had similar subcutaneous fat levels at baseline and were treated with zinc-containing insulin for 6 months. Following this, 7 children were switched to the zinc-free insulin glulisine while the remainder continued zinc-containing insulin treatment, and the intervention group showed improved relative fat thickness (*P*=.003), number (*P*=.01), and size of atrophic sites (*P*=.008) [32].

### Other Skin Manifestations

While most reported insulin-related dermatologic complications fall into the categories described previously, rare cases of more complex pathology also exist. Perez et al [33] describe a case of CSII use leading to inflammatory nodules and friable papules on the upper extremities of a young child. Erosions, subcutaneous nodules, and a pink vascular papule were additionally present on the bilateral buttocks. Biopsy revealed a neutrophilic and granulomatous inflammation at insulin pump injection sites [33]. Switching from CSII to MDI reduced the development of these lesions [33]. Smith et al [34] describe a case of a patient aged 13 years with T1D with previously well-controlled glycemic levels with an HbA_1c_ of 7.2% who developed painful, persistent nodules at all insulin injection sites hours after injection. Following nodule development, the patient’s HbA_1c_ rose to 12.5% [34]. Histopathologic analysis revealed the patient had a panniculitis reaction to exogenous insulin, which was proposed to result from insulin auto-antibodies forming IgG complexes with exogenous insulin, leading to a type III hypersensitivity reaction. Edwards et al [35] report worsening glycemic control paired with inflammatory dermatologic lesions associated with various insulin preparations in a girl aged 17 years. Following negative allergy testing to various insulin prep additives such as zinc, a type III hypersensitivity reaction was determined to be causative [35].

### User Acceptability

User acceptability is crucial in T1D management due to the notable prevalence of adverse cutaneous reactions. Ensuring that devices such as CGM devices and insulin pumps are comfortable and well-tolerated helps maintain consistent use and adherence to treatment regimens. This, in turn, promotes better diabetes control and reduces the risk of complications associated with fluctuating blood glucose levels.

Further, 1 article examined the critical need to reduce “user burden” in diabetes care technology for broader adoption and improved adherence. Surveys of 1348 individuals, including people with diabetes and parents of children with diabetes, highlighted concerns about current CGM devices [36]. Respondents expressed a strong preference for a proposed fully implanted CGM system that eliminates skin-attached components. Specifically, surveys revealed that only 8%‐17% of patients with T1D currently adopt CGM technology, emphasizing the potential of less obtrusive systems to increase usability and adherence rates [36]. These findings underscore the importance of patient-centered design in enhancing diabetes care technologies to achieve broader adoption and better patient outcomes.

In another study involving 67 young patients aged 13 to 19 years with T1D using FGM systems, user acceptability was notably high. The results indicated that 95.5% (64/67) of participants found sensor application less painful than routine finger-stick tests, and 85% (57/67) rated the system as comfortable [37]. Additionally, 94% (63/67) appreciated the small size of the FGM and 89.6% (60/67) felt it did not disrupt their daily activities [37]. The majority (61/67, 91%) reported strong compatibility of the FGM with their lifestyle, and many participants preferred FGM over traditional blood glucose monitoring methods for being less painful (56/67, 83.6%), more discreet (56/57, 83.6%), and easier to use (64/67, 95.5%) [37]. Overall, the study concluded with strong evidence of high acceptability and satisfaction among young patients with T1D using FGM systems.

## Discussion

### Principal Results

Currently, several chemicals are believed to contribute to ACD, including IBOA, butyl acrylate, abietic acid, abitol, and colophony. IBOA is overwhelmingly identified as the causative agent [14,15,17,19-21] and is well known as a causative agent in a variety of these devices. Additional reports exist, identified outside of our original search, whereby a girl aged 8 years developed ACD to IBOA [38], and a case series of both adults and children, in which 4 patients reacted to IBOA and 1 to colophonium [39]. In 2020, IBOA earned the American Contact Dermatitis Society Allergen of the Year title [40]. Manufacturer acknowledgment of IBOA in their devices is mixed, with some companies denying awareness of its presence in their products [41].

Nevertheless, the overwhelming evidence of IBOA as an agent of contact dermatitis should be sufficient to produce consumer warnings and patient transparency. Such allergens often exist on the adhesive [10,14,15,17,19] but have also been found on plastics, plaster, or other aspects of the devices [15,16,19,21,22]. Thus, transparency of chemicals within every component of the various devices is critical to ensure the optimal opportunity to undergo patch testing and prevent adverse dermatologic outcomes. Further, sequiterpene lactone is a co-reactor with IBOA in ACD cases involving diabetic devices and was identified as a causative agent in many of the studies identified in this review [12,18]. This finding illustrates the potential for co-reactivity among devices if a child switches to another device, again prompting the need for increased manufacturer transparency. The overwhelming incidence of contact dermatitis from these devices suggests the need for screening measures for cutaneous complications and patch testing for pediatric patients with T1D to optimize their continued use of these beneficial devices.

Progression of these reactions, such as subsequent infection and long-term scarring, can perpetuate worse outcomes for patients [15,20]. Particularly in toddlers or pediatric patients with less body surface area, minimizing risk and optimizing area availability are potential predictors for ongoing management. In the defining, reviewing, and monitoring skin pathology in T1D study, the authors used noninvasive optical coherence tomography imaging and skin biopsies to identify skin changes in long term CSII users, (average age 48.1, SD 17.1 years). Fibrosis, eosinophilia, increased vessel density, increased IGF-I and TGF-β3, and fat necrosis were identified [42].

Lipodystrophies serve as another barrier to optimizing the use of these devices. Insulin injection pens were identified as having higher rates of lipodystrophies in some studies than continuous insulin pumps, but the reverse was true in others [26,28,29]. Infusion site rotation was determined to be a feasible means of avoiding adverse lipodystrophy reactions, suggesting the need for proper patient education regarding appropriate insulin administration on an individual basis to maintain quality of life regardless of dermatologic complications [26]. Components of insulin formulations are also known to contribute to cutaneous reactions [12,32,34,35,42-44]. It is, therefore, important to identify and isolate reactions from pump components, insulin components, or the nature of a continuous infusion of reaction-provoking insulin. Increased insulin dosage, however, was not found to increase rates of lipohypertroph development [30], suggesting an increased need for studies of the exact cause. Additional potential confounding causative agents must be identified and filtered to better characterize these reactions [45]. Granulomatous reactions were a rare finding in this review, with 2 suggested mechanisms of pathogenesis. First, the altered immune response in T1D and chronic local trauma from insulin injections may lead to a granulomatous tissue reaction. Alternatively, zinc crystals bound to insulin molecules may cause neutrophilic chemotaxis, lysis of those neutrophils leading to enzyme release and further zinc dispersion, and increased chemotaxis in an inflammatory cycle [33,46] Interestingly, the switch to MDI from CSII led to fewer reactions [33], which contradicts the finding of lipodystrophies [26,28].

Identifying effective prevention and maintenance strategies for these cutaneous side effects is critical for patients, parents, and medical providers. Preventing exposure to the offending agents is the primary defense, as effective treatments do not exist to allow for continued use of the products. Colophony was another agent identified in patch testing results, although in this review, it was pertaining to wipes used as a barrier to protect the skin [18,20]. Additional preventative measures identified included silicone-based plasters and hydrocolloid creams, with topical steroids, antibiotics, and emollient creams as therapeutics [24]. The suggested use of barriers such as plasters and adhesives is often cumbersome and requires frequent change, thus decreasing a patient’s tolerance to their usage. Significant cost burdens related to managing these cutaneous effects have been identified as another barrier to continued use. Despite these measures, some patients are still unable to tolerate these effects, leading to discontinued use. Interventions such as laser therapy should be further explored to restore and optimize surface area for device use and insulin administration [31]. A small case series identified topical fluticasone nasal spray prior to CGM application as a successful means of reducing irritation and dermatitis, crediting its anti-inflammatory properties [47].

Additionally, the introduction of a standardized skin reaction report form, as proposed in 1 study, could incentivize health care providers to systematically evaluate and document skin conditions associated with diabetes management devices [48]. This approach holds promise in addressing potential underreporting of adverse events, thereby enhancing the accuracy and comprehensiveness of data collection [48]. By promoting consistent documentation practices, such a tool could yield valuable insights into the prevalence and severity of skin reactions among individuals using these devices. Ultimately, this initiative may contribute to optimizing patient care, informing device selection, and driving advancements in device design aimed at minimizing dermatological complications in diabetes management.

### Limitations

Limitations to this review include confounding variables among insulin length of use, duration of T1D, and unclear manufacturer components. Additionally, some studies had small sample sizes and subjective measurements, often reported by a parent or guardian.

### Conclusion

For pediatric patients with an early age of diagnosis, the lengthened period of need for and exposure to such devices creates an increased risk, and skin reactions contribute as a key reason for treatment discontinuation [49]. Current practices to minimize these cutaneous burdens in pediatric patients include changing site placement, changing devices or brands, and using creams or steroids. Often, these practices are ineffective due to cross-reactivity within the products, high costs, and decreased unaffected surface area with each subsequent cutaneous reaction. These adverse cutaneous reactions can predispose individuals to chronic scarring with psychological sequelae [50]. This review highlights the complex challenges of cutaneous reactions in pediatric T1D patients using insulin infusion and glucose monitoring devices. Increased longitudinal research is required to determine the long-term consequences of discontinued use of the devices and transition to lifelong manual monitoring. Alternative manufacturing practices also need to be considered to optimize patient outcomes. As the current gold standard of insulin-dependent diabetes management depends on continuous devices [50], it is crucial to minimize obstacles to their use and promote lifelong compliance.

## Supplementary material

10.2196/59824 Checklist 1 PRISMA (Preferred Reporting Items for Systematic Reviews and Meta-Analyses) checklist.

## Abbreviations

ACD: allergic contact dermatitis
AD: atopic dermatitis
CGM: continuous glucose monitoring
CSII: continuous subcutaneous insulin infusion
FGM: flash glucose monitoring (device)
IBOA: isobornyl acrylate
MDI: multiple daily injection
PRISMA: Preferred Reporting Items for Systematic Reviews and Meta-Analyses
T1D: type 1 diabetes
